# Targeting Neutrophils for Promoting the Resolution of Inflammation

**DOI:** 10.3389/fimmu.2022.866747

**Published:** 2022-03-16

**Authors:** János G. Filep

**Affiliations:** ^1^ Department of Pathology and Cell Biology, University of Montreal, Montreal, QC, Canada; ^2^ Research Center, Maisonneuve-Rosemont Hospital, Montreal, QC, Canada

**Keywords:** neutrophil, neutrophil trafficking, apoptosis, neutrophil extracellular trap, pro-resolving mediators, GPCRs, resolution of inflammation

## Abstract

Acute inflammation is a localized and self-limited innate host-defense mechanism against invading pathogens and tissue injury. Neutrophils, the most abundant immune cells in humans, play pivotal roles in host defense by eradicating invading pathogens and debris. Ideally, elimination of the offending insult prompts repair and return to homeostasis. However, the neutrophils` powerful weaponry to combat microbes can also cause tissue damage and neutrophil-driven inflammation is a unifying mechanism for many diseases. For timely resolution of inflammation, in addition to stopping neutrophil recruitment, emigrated neutrophils need to be disarmed and removed from the affected site. Accumulating evidence documents the phenotypic and functional versatility of neutrophils far beyond their antimicrobial functions. Hence, understanding the receptors that integrate opposing cues and checkpoints that determine the fate of neutrophils in inflamed tissues provides insight into the mechanisms that distinguish protective and dysregulated, excessive inflammation and govern resolution. This review aims to provide a brief overview and update with key points from recent advances on neutrophil heterogeneity, functional versatility and signaling, and discusses challenges and emerging therapeutic approaches that target neutrophils to enhance the resolution of inflammation.

## Introduction

Acute inflammation is a localized, self-limited, multicellular innate host-defense mechanism against invading pathogens and tissue injury. Polymorphonuclear neutrophil granulocytes play pivotal roles in host defense and are rapidly deployed to the affected sites, where they engage in immediate and intense antimicrobial responses (1–3). Elimination of the offending insult ideally prompts repair of the collateral tissue damage, restoration of tissue function and return to homeostasis (4). However, the neutrophils` powerful weaponry to combat pathogens can cause collateral damage to the host (5). This will then amplifies the initial response through feed-forward inflammatory mechanisms, leading to loss of functional tissue and ultimately to organ dysfunction (6). Neutrophil-driven inflammation is a common mechanism for many diseases, including reperfusion injury, atherosclerosis, cancer, autoimmune diseases, neurodegeneration, and obesity (1, 5, 7). The capacity of neutrophils to augment tissue damage beyond that evoked by the initial infection or tissue injury itself, suggest that early checkpoints control neutrophil kinetics and fate within the inflamed tissue to prevent secondary tissue damage by these effector cells. To assure timely resolution of inflammation, neutrophil influx needs to be stopped, and emigrated neutrophils need to be disarmed and removed from the affected sites.

Neutrophils sense and integrate signals from the inflammatory microenvironment, which modulate their survival and function, and generate cues that can orchestrate innate or adaptive immune effector responses (1, 8). These include secretion of granular proteins (9, 10) cytokines (11), extracellular vesicles (12), neutrophil extracellular traps (13) and formation of membrane tethers (named cytonemes) (14). The role of neutrophils in initiation and progression of a wide range of pathologies makes neutrophils attractive therapeutic targets. However, the critical requirement of neutrophils for antibacterial host defense limits the usefulness of therapies that globally reduce neutrophil numbers or functional responses. Current treatments that target single mediators of inflammation may have limited efficacy because of the redundancy within the innate immune system and many eventually become immunosuppressive (15). Arguably, an ideal therapeutic strategy would be to prevent or reverse neutrophil-mediated tissue injury without impairing their ability to control microbial invasion. One way to develop innovative approaches for the treatment of inflammatory pathologies is to exploit neutrophil biology to enhance the resolution of inflammation. We provide here a brief overview and update with key points from recent advances on neutrophil heterogeneity, functional versatility and signaling, which can be exploited to enhance resolution of inflammation.

## Neutrophils in Homeostasis and Pathogenesis

### Protective Versus Uncontrolled Inflammation

The acute inflammatory response is protective and resolves on their own. Neutrophils are the most abundant leukocytes in blood and form the first line of cellular defense against invading pathogens (1, 2). Neutrophils deploys a potent enzymatic and chemical arsenal to neutralize and clear invaders and necrotic tissues (2, 3) and to facilitate repair (16). Neutrophil trafficking into tissues is a multistep, tightly controlled process (17–19). Aberrant neutrophil recruitment and activation causes tissue damage that amplifies the initial inflammatory response and may continue to chronicity (3, 5, 9).

Preclinical data indicate that impaired neutrophil removal from inflamed tissues results in aggravation and prolongation of the inflammatory responses (5). Accumulating evidence indicates that ongoing inflammation is a prominent component of many diseases, including cardiovascular, acute respiratory, neurodegenerative, metabolic, and autoimmune diseases, arthritis, inflammatory bowel disease, periodontitis and sepsis (7, 18).

The resolution of inflammation is an active process, integrating mechanisms that lead to the restoration of normal tissue function. This process is governed by specialized pro-resolving lipid mediators (lipoxins, resolvins, protectins and maresins), proteins (e.g., annexin A1and galectins) and gaseous mediators (e.g. hydrogen sulfite and carbon monoxide) produced during resolution of self-limited inflammation (15, 20, 21).These mediators act predominantly on phagocytes and other immune cells to instruct repair. Their biosynthesis, receptors, cellular targets, signaling pathways and networks have been described in several excellent reviews (15, 20), and mapped into the searchable Atlas of Inflammation Resolution (21), hence will not be reviewed here. Low grade ongoing inflammation is thought to impair activation of the resolution process (5, 22). Defect in resolution mechanism is increasingly being recognized as an important trigger for acute exacerbation of chronic inflammatory conditions as reported for atherosclerotic plaque rupture (23, 24) or propagation of bacterial infection in mice (25).

While pro-resolving mediators signal through several distinct receptors, two receptors, the β2 integrin Mac-1 (CD11b/CD18) and formyl peptide receptor 2/lipoxin A_4_ receptor (ALX/FPR2) have emerged as master regulators of neutrophil responses and fates.

Mac-1 functions as a bidirectional allosteric “signaling machine” (26). Mac-1 is best known for mediating neutrophil adherence to the activated endothelium and the extracellular matrix (17, 18) and phagocytosis of complement C3b-opsonized bacteria (27). Mac-1 binding to platelets, immune complexes or myeloperoxidase generates survival signals (28), leading to preservation of Mcl-1, the central regulator of lifespan of human neutrophils (29). Phagocytosis of opsonized bacteria induces ROS-dependent activation of caspase-8, which overrides Mac-1 ligation-activated survival signals, resulting in apoptosis (30, 31). Caspase-8 forms a complex with FLIP (FLICE-inhibitory protein), which inhibits RIPK3-dependent necrosis and prevents degranulation (32, 33). Mac-1 also binds neutrophil elastase that directs reverse transendothelial migration (34).

ALX/FPR2 is a member of the formyl peptide receptor family, consisting of three class A G-protein-coupled receptors that share significant sequence homology (35). Formyl peptide receptors recognize pathogen-associated molecular patterns (PAMPs) and damage-associated molecular patterns (DAMPs) to initiate innate immunity. ALX/FPR2 binds an unusually large number of structurally diverse ligands, including proteins, peptides and lipids, and conveys contrasting biological effects (35, 36). For example, ligation of ALX/FPR2 with the acute-phase protein serum amyloid A or the antimicrobial peptide LL-37 activates proinflammatory circuits (37, 38). Annexin A1, annexin A1-derived peptide Ac2-26, lipoxin A_4_, aspirin-triggered 15-epi-LXA_4_ and 17-epi-RvD1 also signal through ALX/FPR2 to limit neutrophil trafficking and lifespan and to promote efferocytosis (25, 39, 40), critical events in the resolution of inflammation. Interestingly, opposing ALX/FPR2 ligands, such as serum amyloid A and lipoxin A_4_, allosterically inhibit each other to bias ALX/FPR2 signaling to promote either inflammation or resolution (37, 41). ALX/FPR2 can form homodimers and heterodimers with FPR1 receptor in ligand-dependent manner, resulting in alternate patterns of downstream signal coupling that dictate neutrophil functional responses (42–45). Recent data suggest that lipoxin A_4_ may act as a biased allosteric modulator, exerting a dual regulatory mechanism on intracellular cAMP accumulation and Ca^2+^ mobilization (45). Binding of serum amyloid A to ALX/FPR2 decreases formation of homodimers and induces phosphorylation of ERK and Akt, whereas lipoxin A_4_ engagement increases heterodimerization with FPR1 with activation of the JNK-caspase-3 pathway, leading to apoptosis in neutrophils (42, 43). While the structural basis of diverse downstream signaling remains largely unexplored, ALX/FPR2 contains a C-terminal motif that mediates receptor recycling following endocytosis and provides protection against apoptosis (46).

Evolving evidence suggest that neutrophils also contribute to wound healing, revascularization and tissue repair (16, 47, 48). Infiltrating neutrophils provide fibronectin as “emergency extracellular matrix” to promote early bone fracture healing (49) and neutrophil-derived matrix metalloprotease 9 facilitates tissue repair in acute lung injury (50). Neutrophil gelatinase-associated lipocalin (NGAL) was reported to orchestrate post-myocardial infarction by increasing the capacity of cardiac macrophages to clear apoptotic cells in mice (51). Conversely, defect in phagocytosis or neutrophil-induced genomic instability in epithelial cells impedes resolution of inflammation and wound healing (12).

### Neutrophil Heterogeneity

Neutrophils are traditionally viewed as a relatively homogeneous cell population with highly conserved function. This perception is, however, rapidly evolving as accumulating data indicate heterogeneity in morphology, phenotype or function under homeostatic and a variety of pathological conditions (52–57). Neutrophil classification has traditionally relied on morphology, gradient separation or surface markers. However, the exact function of some neutrophil subpopulations remain elusive. Single cell RNA sequencing revealed transcriptomically distinct neutrophil populations, even amongst mature peripheral neutrophils (58, 59) and neutrophils associated with chronic inflammatory states (58, 60–62). Different neutrophil states in healthy mice and humans can be projected onto a signal development continuum (termed neutrotime) characterized by clearly defined poles separated by a smooth transcriptome shift (63). Linking the neutrophil transcriptome to the neutrophil phenotype or functional properties will, however, require further investigations. For instance, expression of CD177^+^ (together with membrane-bound proteinase 3) on a subset of human neutrophils facilitates their transmigration (64), hence antimicrobial defense, whereas the elevated frequency of CD117^+^ neutrophils is associated with increased risk of relapse in patients with ANCA-dependent vasculitis (65, 66). VEGF-A recruits a distinct subset of neutrophils with proangiogenic properties into transplanted hypoxic tissues to facilitate restoration of blood supply (67). Reduced CD62L expression on circulating neutrophils defines “senescent” or “aged” neutrophils, which are destined for clearance (68). Neutrophil senescence is controlled by circadian oscillations in the hematopoietic niche (69), the microbiome (70) and the clock-related genes, such as Bmal1 and the CXCR2 signaling pathway (68). Functional heterogeneity, such as competitive phagocytosis (71), in the human circulating neutrophil pool has also been reported, though linking functional responses to phenotype remains challenging.

The widely used term “low density neutrophils” (“low density granulocytes” or “granulocytic myeloid-derived suppressor cells”) also refers to a heterogeneous population of CD66b^+^mature and immature neutrophils with both proinflammatory and immunosuppressive properties (53). Both immature (banded neutrophils) and hypersegmented neutrophils have been identified in this neutrophil subset (53). Hence, the buoyant density of neutrophils is partially coupled to maturation and may rather reflect a spectrum of different densities found in healthy individuals (72). Since mature neutrophils decrease their density when activated *in vitro*, circulating low density neutrophils have been suggested to acquire the activated phenotype within the tissue, perhaps indicating neutrophils that underwent reverse transmigration (72, 73). Consistently, homing neutrophils to the lung was found to switch to an activated phenotype irrespective of the inflammatory disease (74). Low density neutrophils have been implicated in the pathogenesis of systemic lupus erythematosus, albeit it is uncertain whether they are premature neutrophils released from the bone marrow (75) or represent a distinct lineage of neutrophils caused by genomic damage (76). CD10 was suggested as a marker to distinguish proinflammatory and immunosuppressive neutrophils within heterogeneous neutrophil populations in patients with acute or chronic inflammatory diseases (77). A recent study has identified two neutrophil subsets, CD123^+^ immature neutrophils and programmed death-ligand 1 (PD-L1)^+^/CD10^-^ neutrophils as potential biomarkers for patients with sepsis (78). Future studies are needed to elucidate the contribution of these subsets to the pathogenesis of sepsis.

Distinct subsets of tumor infiltrating neutrophils have also been identified. The N1 subset, characterized by hypersegmented nuclei possesses potent tumor killing capacity, whereas the N2 subset that displays an immature phenotype favors tumor growth in mice (79). Although the origin of the N1 and N2 populations is uncertain (80), TGF-β and IFN-β have been implied in polarizing neutrophils toward the N1 phenotype (81, 82). Another subset of tumor-associated neutrophils with antigen-presenting cell features has been found to trigger an anti-tumor T cell response in early-stage of human lung cancer (83). Tumor growth is associated with loss of this neutrophil subset and functional divergence of tumor-associated neutrophils (83, 84).

Mature neutrophils exhibit transcriptional and translational plasticity in response to signals from the inflammatory environment (11) and display a cell-specific pattern of non-coding regulatory regions (85). Thus, *de novo* synthesis of cytokines and membrane receptors, e.g. program death ligand 1 (PD-L1), may alter neutrophil function and contribute to heterogeneity. The importance of gene expression regulation of neutrophils is illustrated by the association of altered methylation profiles with susceptibility to lupus erythematosus (86). Metabolic reprogramming also occurs during the neutrophil life cycle. Neutrophils may utilize glycogen for fuel during phagocytosis or under hypoglycemic conditions (87, 88), and glycogen levels may directly control their lifespan (89). Furthermore, neutrophils exposed to PGE_2_ or PGD_2_ induces a phenotype switch from LTB_4_ production to lipoxin production, which marks the resolution phase (15, 90), further highlighting the functional diversity of these cells.

### Fate of Emigrated Neutrophils

**Figure 1:**
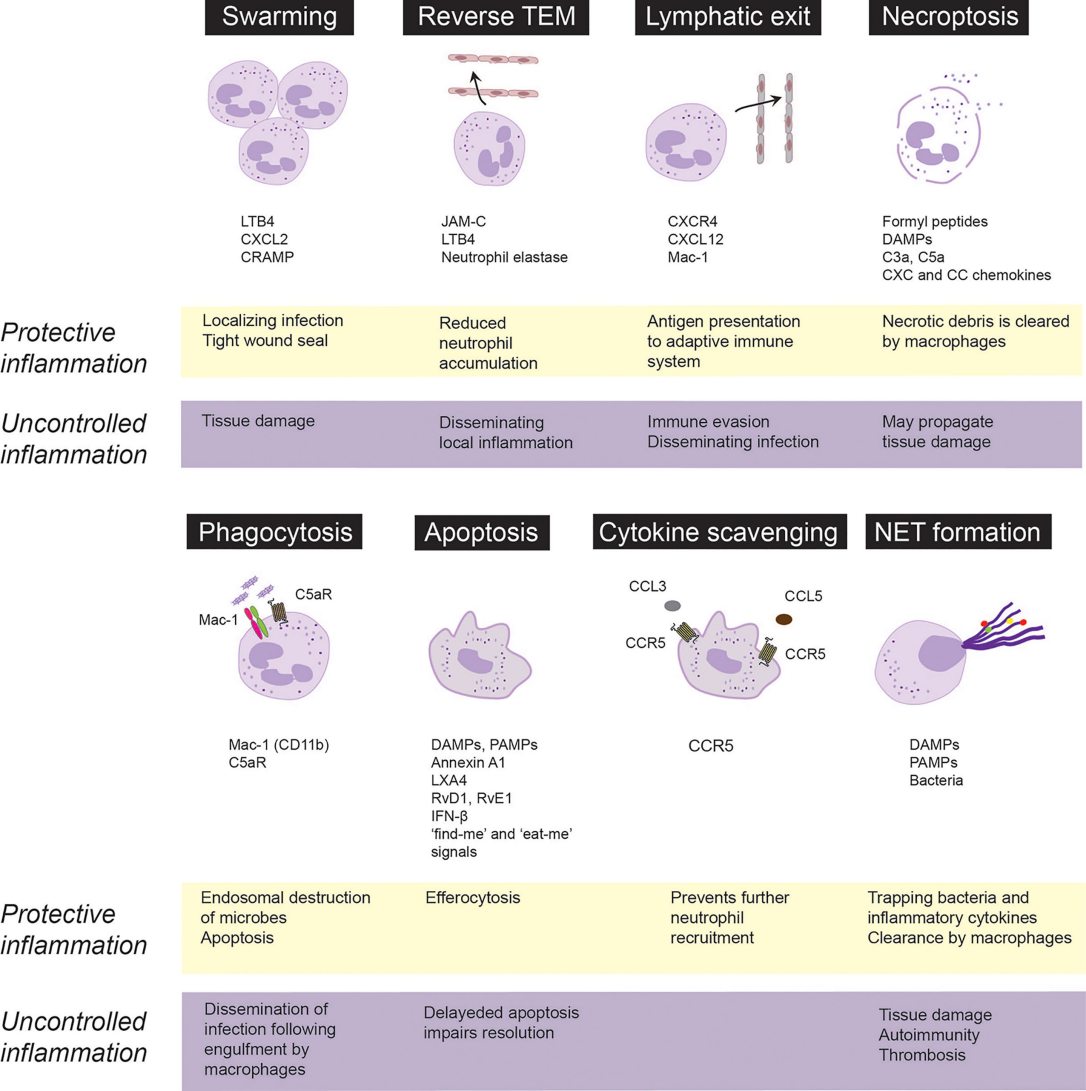
Fate and roles of emigrated neutrophils in protective vs. uncontrolled inflammation. Neutrophils are rapidly recruited from the circulation to the infected or injured tissues. Following extravasation, neutrophils swarm toward the infected sites to localize infection and from a tight wound seal. Neutrophils may trap, neutralize and kill invading pathogens through necroptosis, phagocytosis or release of extracellular traps (NETs). Phagocytosis of opsonized bacteria usually induces apoptosis followed by phagocytosis of apoptotic cells by macrophages *via* efferocytosis. Neutrophils integrate pro-survival and apoptosis-promoting cues from the inflammatory environment, which governs their lifespan. Apoptotic neutrophils express CCR5, which by binding chemokines prevents further neutrophil recruitment. Excessive swarming and necroptosis may aggravate and perpetuate tissue damage. Neutrophils may egress from the inflammatory locus through reverse transendothelial migration (TEM) or lymphatic vessels, which dampen neutrophil accumulation, but may also lead to immune evasion and dissemination of local inflammation. Neutrophils carrying bacteria that they cannot destroy, may serve as “Trojan horses” to disseminate the infection on phagocytosis of apoptotic neutrophils by macrophages. CRAMP, cathelin-related antimicrobial peptide; DAMPs, damage-associated molecular patterns, JAM-C, junctional adhesion molecules C; LTB_4_, leukotriene B_4_; LXA_4_, lipoxin A_4_; PAMPs, pathogen-associated molecular patterns; RvD1, resolvin D1; RvE1, resolvin E1.

Neutrophils recruited to the site of infection or tissue injury engage in different activities to respond to the initial insult, which will also determine that fate and govern their ultimate removal from the inflamed area, critical for protective inflammation and return to homeostasis. By contrast, suppression of certain neutrophil functions or excessive neutrophil responses contribute to uncontrolled inflammation and may lead to chronicity. These responses are discussed in the following sections and summarized on **Figure 1**.

### Neutrophil Swarming

Following transendothelial migration, neutrophils congregate or swarm in tissues, forming clusters around the infected or damaged core to seal off the affected site (90–93). Neutrophil contact with necrotic cells is critical to initiate swarming (94), followed by coordinated LTB_4_ release form neutrophils (91, 95), leading to formation of a stable LTB_4_ gradient that drives concerted waves of neutrophil migration (91, 95). Microscale protein arrays have identified numerous protein mediators, including galectin-3, CXCL8, lipocalin-2 and pentraxin-3that can further enhance LTB_4_-driven neutrophil swarming (95). Mac-1 (CD11b) and LFA-1 (CD11a) mediate neutrophil accumulation in the collagen-free injury center (91). Chemokine receptor trafficking and the LTB_4_ receptor BLT1 coordinate dense neutrophils clusters to form a tight wound seal (91, 96). Development of human neutrophil swarms is associated with lipid mediator class-switching, leading to generation of lipoxin A_4_ and resolvin E3, which, in turn, can limit swarm size (95). By cloaking the injured area (sensing and removing debris as well as damage-associated alarmins), tissue-resident macrophages also contribute to sealing off the damage by preventing initiation of the LTB_4_-driven feedforward signaling cascade that results in neutrophil swarms (94).

Neutrophil swarming has been found to limit tissue damage and contain pathogens in a variety of preclinical models (91, 94, 97, 98), indicating a protective role. Swarming behavior of neutrophils from patients following major trauma or patients receiving immunosuppressive therapy is deficient and is associated with increased susceptibility and reduced ability to clear bacterial (99) or fungal infections (100). Excessive neutrophil swarming leads to collateral tissue damage through release of neutrophil granule content *via* frustrated phagocytosis or necrosis (3, 101) as exemplified by pulmonary ischemia reperfusion injury in mice (97) and inflammation flares around uric acid crystals in gout (102).

### Reverse Transendothelial Migration and Lymphatic Exit

Advances in intravital imaging technologies have revealed that transmigrated neutrophils can also exhibit motility away from inflamed sites, return across the endothelium and re-enter circulation (34, 103–106). Neutrophil reverse transmigration is most prevalent in tissues subjected to ischemia-reperfusion injury (106, 107). Luminal to abluminal transendothelial migration is regulated by various junctional proteins, including VE-cadherin, platelet endothelial cell adhesion molecule-1 and CD99, whereas reverse transmigration predominantly depends on junctional adhesion molecule-C (JAM-C) (106). Under ischemic conditions, excessive production of LTB_4_ induces neutrophil degranulation, expression of neutrophil elastase on Mac-1, which leads to JAM-C degradation and reverse transmigration (34). Reverse transmigrated neutrophils display a different phenotype, characterized by high ICAM-1 and low CXCR1 expression, increased capacity to produce superoxide and prolonged lifespan (103, 106), thereby contributing to the heterogeneity of circulating neutrophils. The biological consequences of reverse transmigration are unclear and may depend on the circumstances. As reverse transendothelial migration leads to removal of neutrophils from the inflamed site, it may function as a protective mechanism that limits the inflammatory response (104, 108). On the contrary, reverse transmigration could lead to systemic propagation of inflammation or distant organ injury in mice subjected to cremaster muscle or lower-limb ischemia-reperfusion injury (34, 106).

An alternative way of neutrophil egress from the inflamed site may involve the exit through the lymphatic vessels. During infection, neutrophils were detected carrying living bacteria from the infected tissue to draining lymph nodes in mice (109–112). Skin egress of neutrophils *via* lymphatic vessels depends on CXCR4 and its ligand CXCL12 expressed by lymphatic endothelial cells as well as on Mac-1 (111, 113), though the counter-ligand for Mac-1 remains to be identified. *Staphylococcus aureus*-pulsed neutrophils recruited into the lymph node acquire the phenotype (expression of major histocompatibility complex (MHC) II and the costimulatory molecules CD80 and CD86) and functionality of antigen-presenting cells to initiate adaptive immunity (111, 114). By contrast, other studies have proposed that neutrophils carrying bacteria or viruses may lead to immune evasion and permit the dissemination of the infection upon engulfment by macrophages (115, 116).

### Neutrophil Lifespan, Apoptosis and Efferocytosis

Mature neutrophils have a short half-life in the circulation (117, 118) and die rapidly *via* apoptosis (119). Following recruitment to inflamed tissues, neutrophil lifespan is increased through delaying apoptosis in response to PAMPs, DAMPs and environmental signals, though the extent of increased lifespan remains unknown (120, 121). Neutrophils contribute to interstitial acidosis, which serves as a danger signal (121) that extends neutrophil lifespan by preserving the expression of the anti-apoptotic protein Mcl-1 (122). Hypoxia or bacterial infections even under normoxia were shown to induce release of HIF-1α and HIF-2α, which generates survival cues for neutrophils and enhances their bactericidal activity to restrict systemic spread of infection (123, 124). Activated neutrophils release myeloperoxidase that activates a Mac-1-centered feed-forward loop to induce degranulation and generate survival signals, thereby perpetuating the inflammatory response (28). Conversely, genetic deletion of myeloperoxidase or disruption of the myeloperoxidase-triggered feedforward loop with 15-epi-LXA_4_ limits neutrophil-evoked tissue damage and facilitates resolution (40, 125, 126).

Extended neutrophil lifespan through delayed apoptosis is a common feature of many inflammatory diseases, including sepsis (127, 128), acute respiratory distress syndrome (129), severe asthma (130) and acute coronary syndrome (131), and is associated with disease severity. In experimental models, suppressing neutrophil apoptosis prolongs and aggravates the inflammatory response (28, 132), whereas promoting neutrophil apoptosis with cyclin-dependent kinase inhibitors (133), 15-epi-LXA_4_ (40), or IFN-β (134) accelerates the resolution of inflammation. Consistently, genetic deletion of the pro-apoptotic ARTS protein hinders the execution of the intrinsic apoptosis program in neutrophils and delays activation of resolution programs (135).

Phagocytosis of complement-opsonized bacteria or necrotic cells overrides survival signals generated by Mac-1 ligation and accelerates neutrophil apoptosis (also known as phagocytosis-induced cell death or PICD) (30, 31). Complement-mediated phagocytosis is governed by a delicate balance between Mac-1 and the complement C5a receptor (C5aR or CD88) (136, 137). Thus, reduced Mac-1 expression or genetic deletion of C5aR disables phagocytosis and reduces bacterial killing (136, 138). Bacterial or mitochondrial DNA signaling through TLR-9 upregulates Mac-1 expression and induces neutrophil elastase and proteinase 3-mediated shedding of C5aR, leading to reduced phagocytosis of *E. coli*, suppressed PICD and efferocytosis, thereby prolonging acute lung injury in mice (25). Conversely, by preventing TLR9 activation-mediated Mac-1 upregulation and C5aR shedding, aspirin-triggered 15-epi-LXA_4_ and 17-epi-RvD1 restore the balance between Mac-1 and C5aR and consequently enhance phagocytosis, bacterial killing, PICD and the resolution of lung injury (25). Of note, the pro-resolving lipid mediators resolvin E1 and resolvin D5, which signals through the LTB_4_ receptor BLT1 (31) and GPR32 (139), respectively, can also enhance phagocytosis of bacteria by naïve neutrophils.

Removal of apoptotic neutrophils (and other cell types) by macrophages is critical for restoring tissue homeostasis. The detection and elimination of apoptotic cells are orchestrated by “find-me” and “keep-out” signals that regulates recruitment of phagocytes to the vicinity of apoptotic cells and “eat-me” signals that allow recognition and engulfment (139–143). Apoptotic cells release nucleotides, such as ATP and UTP through caspase-mediated activation of pannexin 1 channels, which act as key “find-me” signals (144, 145). By contrast, lactoferrin released by apoptotic cells inhibits neutrophil chemotaxis without hindering monocyte recruitment (146), thereby assuring the recruitment of appropriate phagocytes for clearance of apoptotic cells and limit inflammation. Efferocytosis induces a metabolic switch in engulfing macrophages, leading to glycolysis and lactate release through SLC16A1 and reprograms macrophages from the inflammatory phenotype to an anti-inflammatory phenotype (22, 147, 148) and subsequently to a CD11b^low^ subset with minimal phagocytic activity, increased oxidative phosphorylation and expression of IFN-β-related gene signature (134, 149). By promoting neutrophil apoptosis and efferocytosis as well as reprogramming macrophages to the CD11b^low^ phenotype, IFN-β orchestrates bidirectional cross-talk between neutrophils and macrophages to accelerate resolution (134). Genetic deletion or pharmacological inhibition of cyclin-dependent kinases 5 and 9 drives neutrophil apoptosis and reprograms macrophages, thereby facilitating neutrophil clearance and resolution (133, 150).

Neutrophils carrying *Toxoplasma gondii* or *Leishmania donovani*, which they cannot destroy, may serve as “Trojan horses” to disseminate the infection following macrophage engulfment (115, 116). The Gram-negative intracellular coccobacillus *Francisella tularensis* can evade phagosomal elimination and replicates in the cytosol (151) parallel with sustaining mitochondrial integrity and delaying neutrophil apoptosis (152, 153). Continued accumulation of dysfunctional neutrophils at the infection site is thought to contribute to disease exacerbation.

### NETosis

Among the neutrophil defense armory is the release of extracellular traps (NETs), consisting of a nucleic acid scaffold decorated with histones and granular proteins to entrap and kill bacteria, viruses and fungi (13, 154, 155). Suicidal NET release (commonly referred to as NETosis) occurs in response to various stimuli and classically involves activation of protein kinase C and the Raf-MEK-ERK pathway, NADPH-dependent translocation of neutrophil elastase and myeloperoxidase from cytosolic granules into the nucleus, leading to the breakdown of chromatin and the nuclear envelop. NETs are extruded following the rupture of the neutrophil cell membrane (13). Hence, NETosis may be considered as a distinct form of necrotic cell death (156). Differences in NET composition have also been reported (157, 158), though the implications of these differences remain to be investigated. NET release may also occur in the absence of cellular suicide (also known as vital NETosis) in response to recognition of certain bacteria or PAMPs (154). For example, HMGB1 released from activated platelets or necrotic cells evokes NET release through interactions with TLR4, independent of NADPH oxidase (159), while suppressing phagocytosis (160). Vital NETosis requires vesicular trafficking of DNA for delivering the NET out of the cell without requiring membrane perforation (161). NET caused by extrusion of mitochondrial rather than nuclear DNA does not cause lytic cell death (162). Reports also exist that neutrophils that had already underwent vital NETosis were still capable of chasing and imprisoning live *Staphylococcus aureus* or *Candida albicans*, whereas NETs recruited additional neutrophils in a swarming-like behavior (161, 163). Although limited information is available on the molecular switches that trigger phagocytosis, NETosis or degranulation, it is plausible that selective activation of these processes assures the most effective neutrophil response to an insult. One possible control mechanism is ALX/FPR2, as genetic deletion of Fpr2 (the equivalent of human ALX/FPR2) in mice is associated with excess NET production and more severe lung injury following bacterial infection (164).

NETs are eventually degraded by macrophages and dendritic cells through DNase 1 that cleaves chromatin within NETs (165) or the cytosolic exonuclease TREX1 (DNase III) following endocytosis (166, 167). The antibacterial protein LL-37 facilitates NET uptake by macrophages, while protecting NETs against degradation by bacterial nucleases (167). A recent study reported that the thirteen-series (or T-series) resolvins, present in resolution exudates, enhance NET uptake by macrophages through the cAMP-PKA-MAPK pathway (168). The receptor for T-series resolvins remains to be identified.

NETs effectively capture a large range of microbes, exert direct antimicrobial activities and demarcate the infected locus (169, 170). However, since the effects of NET components are not restricted to invading pathogens, excessive or uncontrolled NET formation can inflict damage to the surrounding tissue, maintaining a pro-inflammatory and pro-thrombotic environment that underlies various pathologies. For example, extracellular histone components through TLR-mediated generation of thrombin can evoke microaggregation, and endothelial and tissue injury (169, 171, 172), whereas neutrophil granule constituents expressed on NETs, such as proteinase 3 or myeloperoxidase can trigger autoimmunity when NET degradation is impaired (165, 173). Clinical studies have also reported an association between NET generation and disease severity in sepsis-induced (164, 174) or COVID-19-associated acute respiratory distress syndrome (175–177). Albeit wide-ranging differences in intrapulmonary neutrophils were reported in COVID-19 autopsies (178), intense neutrophilic inflammation and NET release contribute to progression of the disease and higher mortality (175, 179–181). NETs infiltrate the airways, pulmonary interstitial space and vasculature in severe COVID-19 (182), leading to tissue damage and formation of microthrombi in pulmonary capillaries (175, 177, 179–181). Activated neutrophils secrete ROS and proteases, which in turn, enhance NETosis and inactivate plasma antiproteases that protect against neutrophil proteases (183). These create a vicious cycle to propagate tissue destruction. Enhancing NET degradation by DNase I or partial genetic deletion of peptidyl arginine deiminase 4 (PAD4^+/-^) reduced the severity of bacterial lung injury in mice (164). Complete PAD4 deficiency markedly suppressed NET formation and lung injury, but increased bacterial burden, indicating a shift in the balance between the protective and deleterious actions of NETs during bacterial infections.

Accumulating evidence indicates dual role for NETs in cancer (184). NETs were found to inhibit proliferation of colon carcinoma cells (185) and exert cytotoxic activity on malignant melanoma cells (186). Accumulation of NET producing CD16^high^ CD62L^dim^ neutrophils in tumor sites was reported to predict improved survival in patients with head and neck squamous cell carcinoma (187). In contrast, tumor cells can prime neutrophils to release NETs (188), forming an amplifying loop that links NET formation to tumor progression. As an example, NET-associated neutrophil elastase and matrix metallopeptidase 9 (MMP9) could awaken dormant cancer cells, thereby promoting invasion and metastases (189, 190). NETs may shield tumor cells against NK cells and cytotoxic T cells (191), exert pro-angiogenic activities that support tumor growth (192), and contribute to tumor-associated thrombosis (193, 194) and hypercoagulability (195). Furthermore, NETs can also capture tumor cells and carry them in the circulation, thus favoring tumor dissemination (196). NET-DNA was also shown to act as a chemotactic factor to attract tumor cells through binding to the transmembrane protein CCDC25 expressed on primary cancer cells, thereby promoting metastasis (197).

### Necrosis and Necroptosis

At sites of inflammation, neutrophils can undergo necrotic cell death, which occurs in a disorderly manner following cell injury, or necroptosis, a programmed form of necrosis (198). Necrotic cell death is associated with the release of DAMPs and cell debris, which are potent inducers of inflammation (198). TNFα or ligation of the adhesion receptors Mac-1, CD18, CD15 or CD44 in GM-CSF-primed neutrophils activates the receptor-interacting protein kinase 1 (RIPK1)-RIPK3- mixed lineage kinase domain-like protein (MLKL) signaling pathway (199, 200), leading to translocation of MLKL1 to the inner leaflet of plasma membrane and membrane permeabilization (201). X-linked IAP (XIAP) ubiquitinylates RIPK1 (202) and thus functions as a switch to direct neutrophils to either necroptosis or apoptosis (201). NADPH oxidase-mediated generation of ROS is essential for necroptosis (199, 200). Consistently, neutrophils from patients with chronic granulomatous disease (caused by a genetic defect in NADPH oxidase) do not undergo necroptosis (200). Some studies reported association of necroptosis with NET formation in a mouse model of gouty arthritis (203), though NET release occurred independently of RIPK3 and MLKL signaling (204). Phagocytosis of methicillin-resistant *Staphylococcus aureus* redirects neutrophils from phagocytosis-induced apoptosis to necroptosis, which may allow the escape of viable bacteria from dead neutrophils, thereby persisting infection (205, 206). This requires RIPK3, but not RIPK1 and MLKL, and is associated with RIPK3- and protease-mediated production of IL-1β (205, 207). Neutrophil necroptosis, evidenced by the activation of RIPK3 and MLKL, was detected in tissue samples from patients with neutrophilic diseases (200). The pathological significance of these observations remains elusive.

Dying cells release DAMPs, including mitochondrial formyl peptides, purines, LTB_4_, cytokines and chemokines, and triggers generation of C3a and C5a, which collectively function as “find-me” signals for phagocytes (208–210). Similar to apoptotic cells, necrotic cells also express “eat-me” signals, such as externalization of phosphatidylserine and LTB_4_, which facilitate their clearance by macrophages (209). “Eat-me” cues unique to necrotic cells include deposition of complement C1q on the cell membrane (211) and cell surface externalization of annexin A1 (212). The importance of removing necrotic neutrophils is illustrated by the role of the neutrophil granule constituent proteinase 3 in autoimmune vasculitis (65, 66) and chronic obstructive pulmonary disease (213).

## Therapeutic Opportunities

**Figure 2:**
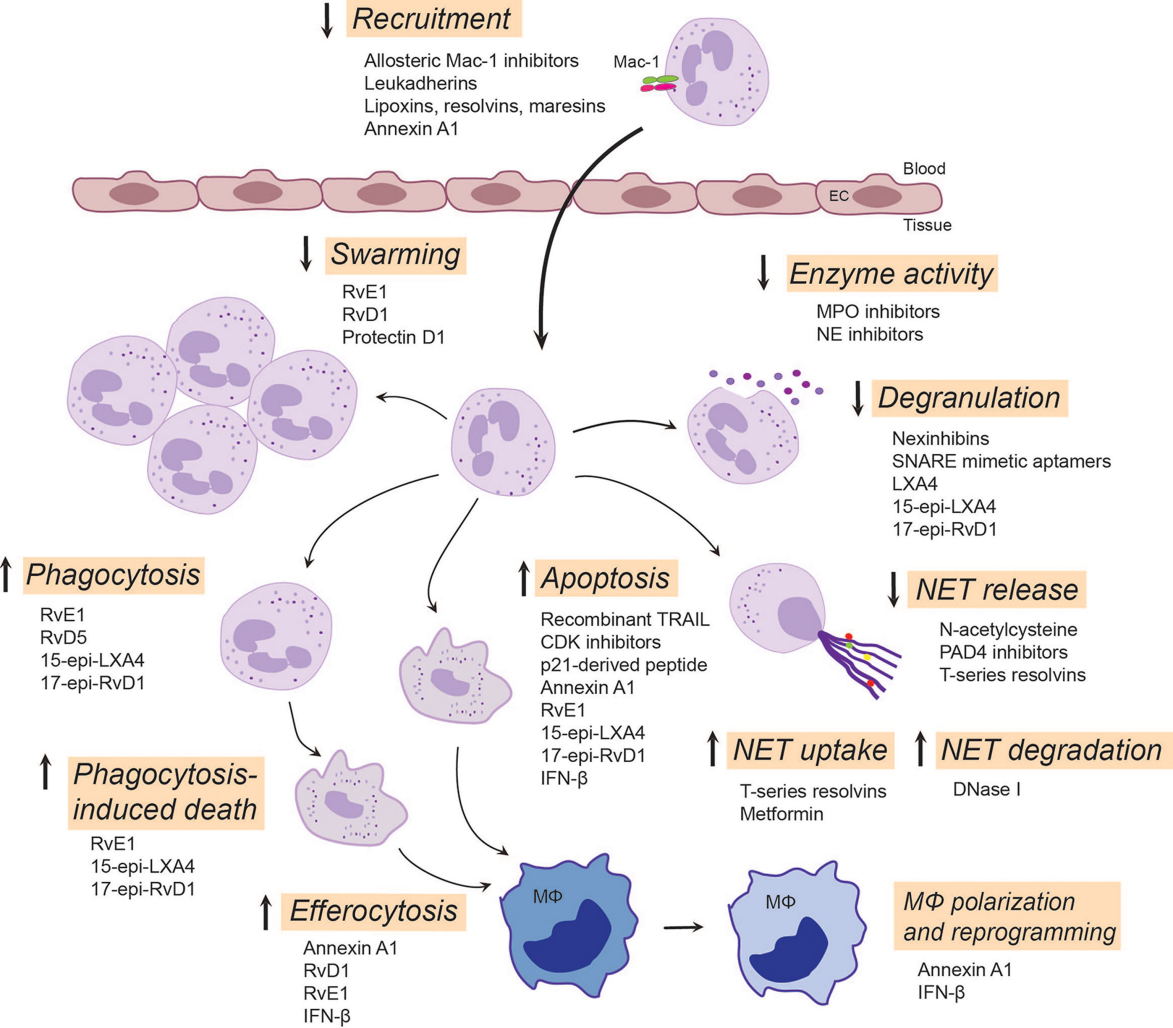
Emerging neutrophil-targeted therapeutic approaches to promote the resolution of inflammation. The strategies include blocking, restoring or activating neutrophil functions. Thus, blocking function of Mac-1 or upregulation of Mac-1expression dampens neutrophil accumulation, a critical component of terminating the inflammatory response. LXA_4_, RvE3 and protectin D1 serve as stop signals for swarming. Inhibition of degranulation or the activity of secreted enzymes, such as MPO and NE, could reduce tissue injury and alter composition of NETs. Enhancing NET degradation by DNase I or promoting NET uptake by T-series resolvins or metformin may prevent the deleterious actions of excessive NET formation. RvD5 and RvE1 facilitates phagocytosis, whereas 15-epi-LXA_4_ and 17-epi-RvD1 restore impaired phagocytosis, facilitate clearance of bacteria and phagocytosis-induced apoptotic cell death. By countering survival cues, many molecules, including CDK inhibitors, annexin A1, IFN-β and lipid SPMs, can redirect neutrophils to apoptosis and promote their uptake by macrophages through efferocytosis. This leads to reprogramming and polarization of macrophages toward a pro-resolution, regenerative phenotype that promotes further removal of neutrophils. Annexin A1, RvE1, RvD1 and IFN-β play pivotal roles in mediating feedforward resolution programs. Of note, although most of these data are from experimental models, some strategies (e.g. LXA_4_ mimetics, NE inhibitors or DNase I) are currently being investigated in clinical trials. C5aR, complement C5a receptor; CDK, cyclin-dependent kinase; EC, endothelial cell; IFN-β, interferon-β; LXA_4_, lipoxin A_4_; 15-epi-LXA_4_, 15-epi-lipoxin A_4_; MPO, myeloperoxidase; NE, neutrophil elastase; NET, neutrophil extracellular traps; PAD4, peptidyl arginine deiminase 4; 17-epi-RvD1, 17-epi-resolvin D1; RvE1, resolvin E1; RvE3, resolvin E3; RvD5, resolvin D5; SNARE, soluble N-ethylmaleimide-sensitive-factor attachment protein receptor; SPMs, specialized pro-resolving mediators; TRAIL, TNF-related apoptosis-inducing ligand.

Given the central role of neutrophils in inflammation, it is paramount to seek novel therapeutic approaches controlling neutrophil-mediated collateral tissue damage and/or facilitating clearance of neutrophils from the inflamed site upon fulfillment of their immediate mission. Indeed, a variety of strategies have been developed to prevent the detrimental effects of neutrophils, with some approaches entering clinical trials (**Figure 2**).

### Beta-2 Integrin-Targeted Therapeutic Approaches

Mac-1 conformations and broad ligand recognition specificity shape neutrophil responses and contribute to neutrophil functional heterogeneity (30, 214). Hence, β2 integrins have attracted considerable interest as potential therapeutic targets. Indeed, currently available monoclonal antibodies and small molecule inhibitors that block the ligand-binding site and a broad repertoire of β2 integrin functionality efficiently reduced neutrophil-driven inflammation in numerous experimental models (7, 18, 214). However, global β2 integrin blockade lacks functional selectivity, and can impair phagocytosis and antibacterial defense (215). Conventional β2 integrin blockade may also increase the risk of development of LAD-like symptoms. Alternative approaches include targeting Mac-1 conformation or ligand-specific signaling mechanisms without compromising host defense. Selective inhibition of Mac-1 binding of its ligand CD40L with the M7 monoclonal antibody reduced inflammation without affecting protective immunity (19, 216). Allosteric inhibitors that stabilize β2 integrins in the high affinity bent conformation efficiently blocked neutrophil adherence (217) and restricted neutrophil accumulation in murine models (218, 219). Selective targeting of discrete glycan motifs present on Mac-1 with plant lectins was reported to reduce neutrophil adhesion and trans-epithelial migration, while enhancing phagocytosis and neutrophil apoptosis (220). Other studies have reported that activation of Mac-1 with the small molecule agonists leukadherins reduced neutrophil trafficking into the kidney, while augmented leukocyte adherence to the endothelium in murine models (221). These resulted in attenuation of arterial narrowing and improved renal function. Leukadherin-1 was reported to activate microRNA Let7a and induce polarization of M0 macrophages toward the pro-inflammatory M1 phenotype that drives anti-tumor immunity (222). Thus, leukadherins likely exert context-dependent actions. Hence, additional studies are required to explore their effects on macrophage polarization and the resolution of inflammation.

Among the mechanisms by which specialized pro-resolving mediators (SPMs) facilitate resolution of inflammation is inhibition of neutrophil trafficking into the inflamed site by decreasing their adhesion and transmigration. SPMs signal through stereospecific binding to cellular receptors (223) to prevent upregulation of Mac-1 expression on neutrophils and to reduce Mac-1-mediated neutrophil adhesion and transendothelial migration, consequently limiting their tissue accumulation (15, 21). Thus, annexin A1, lipoxin A_4_ and resolvin D1 interact with ALX/FPR2, resolvin E1 binds ERV1 and resolvin D2 binds to DRV2 to repress Mac-1 expression (20, 223). In a feedforward mechanism for resolution, SPM receptor signaling by one mediator can trigger mobilization or synthesis of other SPMs for other receptors, exemplified by LXA_4_ mobilization of annexin A1 to limit neutrophil trafficking into the inflamed microvasculature (224), resolvin D1-triggered LXA_4_ generation in periodontal wound healing (225), and resolvin E1-ERV1-induced biosynthesis of LXA_4_ for ALX/FPR2-mediated resolution of allergic lung inflammation (226). Of note, the gaseous mediator hydrogen sulfide and mast cell-stabilizing drug nedocronil also mobilize annexin A1 to control leukocyte trafficking in the mouse mesenteric circulation (224, 227).

### FPR2 Agonists: Shifting the Balance Towards Resolution

As the pleiotropic receptor ALX/FPR2 conveys ligand-specific pro- or anti-inflammatory actions, it has been proposed to function as a master switch to initiate the resolution of inflammation. A unique feature of ALX/FPR2 is that ligation of this receptor can to activate several, if not all of the processes that are critical for inflammation resolution, including blocking neutrophil trafficking into tissues, promoting neutrophil apoptosis and macrophage efferocytosis (15, 20, 25, 31, 40). As an example, annexin A1 and its mimetic peptide Ac2-26 induce the detachment of adherent neutrophils from the endothelium and inhibit neutrophil chemotaxis, thereby controlling neutrophil accumulation within the inflammatory locus (228), regulate phagocytosis of bacteria and fungi (229) and accelerate neutrophil apoptosis (39). Annexin A1 released from apoptotic neutrophils recruits monocytes to clear apoptotic cells (230), promotes their polarization towards the M2 phenotype (231), thereby protecting the surrounding healthy tissue and accelerating muscle regeneration through AMPK activation (232). Activated neutrophils releases annexin A1-containing microparticles and exosomes, which mediate its anti-inflammatory activity (233) and orchestrate epithelial wound repair through ALX/FPR2 and FPR1 (234). Neutrophil-derived microvesicles can enter cartilage and protect the joint in inflammatory arthritis (235). These findings raise the possibility of harnessing annexin A1-loaded microvesicles as a therapeutic strategy for reducing neutrophil infiltration and protection against tissue damage.

Ligation of ALX/FPR2 with LXA_4_, resolvin D1 and aspirin-triggered 15-epi-LXA_4_ and 17-epi-RvD1 activates mechanisms that partially overlap those stimulated by annexin A1, but also distinct patterns of activation for intracellular pathways, including ERK and NF-κB phosphorylation (15, 31). In addition to ALX/FPR2, to date, three other surface receptors, ERV1, DRV1 and DRV2 have been identified mediate cell-specific actions of SPMs (15, 223). A common feature of ligation of these receptors is attenuation of neutrophil activation and trafficking into inflamed tissues (15, 20, 223). SPM binding to ALX/FPR2 decreases NF-κB activity and cytokine production (31, 236), disrupts the myeloperoxidase-centered feedforward loop and redirects neutrophils to apoptosis (40), and restores TLR9-impaired phagocytosis of bacteria and promotes phagocytosis-induced neutrophil death (25). Consistently, 15-lipoxin A_4_ was found to accelerate the resolution of inflammation in a variety of experimental models, including asthma (237), peritonitis (238, 239), cystic fibrosis (240), ischemia-reperfusion (139), and myeloperoxidase and *E. coli*-induced acute lung injury in mice (25, 40). By reducing bacterial burden, ALX/FPR2 agonists may also be used to lower reduce antibiotic requirements or used in conjunction with antibiotics to strengthen host defense against infections (241, 242). Most SPMs are metabolically inactivated within the inflammatory site, some SPMs reach circulation (242, 243). To circumvent rapid inactivation, several metabolically stable analogs, such as benzo-RvD1, were synthesized (15) and nanomedicines were designed to deliver SPMs and their analogs to promote wound healing (244). Furthermore, the lipoxin A_4_ analog BLXA4-ME is currently in trial for periodontal inflammation (245), whereas other molecules are in clinical development program (15). Nanomedicine delivery of SPMs to correct resolution deficits represents a fascinating novel avenue for preventing the progression of chronic diseases, as exemplified by the decreases in tissue SPMs immediately before plaque rupture (23, 24).

The intriguing biology of the ALX/FPR2 receptor has initiated numerous medicinal chemistry programs to develop small-molecule agonists to activate resolution programs (15, 246). Relevant examples here are the beneficial actions of synthetic lipoxin mimetics and the prototype peptide agonist WKYMVM in various preclinical models (247, 248). Phase I clinical trials reported promising tissue protective actions with other small-molecule ALX/FPR2 agonists, such as compound ACT-389949 (Actelion) (249), and compound BMS986235 (Bristol –Myers Squibb) (250) in heart failure. However, since ALX/FPR2 expression is not restricted to myeloid cells, further studies are required to identify the cellular targets (e.g. endothelium, smooth muscle cells or fibroblasts) mediating the beneficial actions of these compounds.

### Targeting Neutrophil Lifespan and Apoptosis

Several preclinical studies indicate the therapeutic potential of targeting neutrophil apoptosis for facilitating the resolution of inflammation. Thus, pharmacologic blockade of cyclin-dependent kinases (CDKs), which principally inhibit CDK9-mediated transcription of Mcl-1 (150, 251) has been shown to exert potent anti-inflammatory effects in experimental models of neutrophil-dominated inflammation and enhance resolution of severe lung injury models (133, 251, 252). Interestingly, the CDK inhibitor drug R-roscovitine also increased bacterial clearance (251) through a yet unidentified mechanism. *Ex vivo* studies showed that the CDK inhibitor AT7519 efficiently overrides the delayed neutrophil apoptosis in patients with sepsis-associated ARDS concurrent with reduced expression of Mcl-1 (253). Studies in preclinical models indicate that several SPMs, including 15-epi-lipoxin A_4_ and resolvin E1, signaling through ALX/FPR2 and the LTB_4_ receptor BLT1, respectively, can also override pro-survival cues and redirect neutrophils to apoptosis in part by reducing Mcl-1 expression (31, 40). In line with these observations, the annexin A1 mimetic peptide Ac2-26 induces neutrophil apoptosis (254, 255). Furthermore, IFN-β, produced by resolution phase macrophages, drives neutrophil apoptosis through the IFNαR1-STAT3 signaling pathway and acceleration of Mcl-1 degradation (134). These findings identify Mcl-1as a promising target for resolution therapy.

Another potential mechanism to accelerate neutrophil apoptosis is restoring impaired phagocytosis. Indeed, bacterial and mitochondrial DNA were shown to reduce phagocytosis and consequently bacterial clearance as well as phagocytosis-induced death by inducing the cleavage of complement C5a receptor, which acts in concert with Mac-1 to mediate phagocytosis (10, 256). By preventing cleavage of C5a receptor, aspirin-triggered 15-epi-lipoxin A_4_ and 17-epi-resolvin D1 restore impaired phagocytosis, enhances bacterial clearance, drive phagocytosis-induced death and consequently attenuate *E. coli*-evoked lung injury in mice (25).

Other strategies to modulate neutrophil apoptosis include activating the extrinsic pathway of apoptosis by TNF-related apoptosis-inducing ligand (TRAIL) and the use of peptides derived from the cyclin-dependent kinase inhibitor p21. While TRAIL appears to have no role in constitutive neutrophil apoptosis, treatment with recombinant TRAIL was shown to enhance neutrophil apoptosis and limit the inflammatory response to LPS in mice (257). The p21 peptide binds and sequesters proliferating cell nuclear antigen (PCNA), which acts as a cytoplasmic platform to control the lifespan of human neutrophils (258). Consistently, a p21-derived peptide was shown to induce apoptosis in neutrophils isolated from patients with *Pseudomonas aeruginosa* infection (259), highlighting PCNA as a novel target to modulate pathological inflammation. Apoptotic neutrophils and T cells sequester chemokines, such as CCL3 and CCL5, through modulation of CCR5 expression, thereby reducing the availability of proinflammatory cytokines for other neutrophils and preventing further neutrophil recruitment (260). An interesting approach emerged from these observations is that administration of apoptotic cells markedly reduced the cytokine/chemokine storm and protected against acute lung and kidney injury in a mouse model of severe sepsis (261).

Macrophages play a crucial role in the clearance of apoptotic and necrotic cells, including neutrophils (140, 209, 262). Various pathologies, including ARDS is associated with impaired macrophage phagocytic function (263, 264). Thus, restoring macrophage function represents another avenue of potential therapy. As an example, IFN-β, produced by resolution phase macrophages, mediates a feedforward loop to promote neutrophil apoptosis and efferocytosis, which contributes to macrophage reprogramming and production of additional IFN-β (134). Several clinical trials reported favorable response to early IFN-β use to mitigate SARS-CoV2 infection-associated severe ARDS and other studies are underway testing the clinical efficacies of type I interferons (265).

### Modulation of Degranulation, NET Release and Clearance

As excessive or aberrant NET formation has been implicated in the pathogenesis of many pathologies, inhibiting NET release or enhancing NET clearance open promising avenues for therapy. Preclinical studies showed that ROS scavengers, such as N-acetyl cysteine (266), myeloperoxidase inhibitors (267) and PAD4 inhibitors (268–270) could inhibit NET release and dampen tissue injury in experimental models of arthritis, arteriosclerosis and autoimmune diseases. Likewise, the reversible PAD4 inhibitor GSK484 was shown to inhibit suicidal NETosis (271) and to prevent cancer-associated neutrophil-mediated renal injury in mice (272). Studies in PAD4-knockout mice suggest that bacterial infections may shift the balance of the protective and deleterious effects of NETs in host defense (273, 274). Select lipid SPMs, such as resolvin D4 (275) and T-series resolvins (168) also limit NET formation, though the underlying molecular mechanisms are incompletely understood.

Another potential therapeutic approach is blocking neutrophil degranulation or the effects of granule enzymes. Neutrophil-specific exocytosis inhibitors, termed Nexinhibs, and SNARE domain-derived peptide aptamers have been developed. Nexinhibs selectively inhibit release of azurophil granule contents by interrupting the Rab27a-JFC1 interaction without affecting phagocytosis (276, 277), and reduce neutrophil accumulation in the kidney and liver in a mouse model of systemic inflammation (277). Intrapulmonary delivery of Nexinhib20-loaded nanoparticles, which release Nexinhib20 upon cleavage by neutrophil elastase, was shown to dampen neutrophil recruitment and degranulation within the lower airways (278). SNARE mimicking peptide aptamers exhibit varying selectivity towards neutrophil granule subsets (276). As an example, TAT-SNAP-23 (a fusion protein containing the N-terminal SNAP-23 SNARE domain fused with the cell penetrating HIV peptide TAT) was reported to attenuate lung injury evoked by pulmonary immune complex deposition (279) or sepsis (280). However, since SNARE expression is not restricted to neutrophils, further studies are needed to distinguish their actions of neutrophils and other cell types *in vivo*. Several SPMs, including lipoxin A_4_, resolvin D1 and aspirin-triggered 15-epi-LXA_4_ and 17-epi-RvD1, acting through ALX/FPR2, also block myeloperoxidase- or TLR9 activation evoked release of myeloperoxidase, neutrophil elastase and proteinase 3 and consequently accelerate resolution of sterile (40) and *E. coli*-induced lung injury in mice (25). Several synthetic inhibitors and natural compounds became available over the past years, in particular compounds that target neutrophil elastase (281). Neutrophil elastase inhibition was reported to prevent progression of lung injury in various experimental models (282). However, the synthetic selective neutrophil elastase inhibitor, sivelastat failed to improve 28-day mortality in patients with ARDS (283). It remains to be investigated whether this was due to its toxic or off-target effects.

Promoting NET degradation by treatment with DNase I attenuated tissue injury and increased survival in mouse models of severe bacterial pneumonia/acute lung injury (164, 174), transplantation-associated lung injury (284), tumor (285) and lupus (286). Furthermore, an ongoing phase III clinical trial is investigating the effectiveness of inhaled dornase-α (recombinant human DNase I) in reducing the incidence of ARDS in severe trauma patients (287). Other bioengineered DNases, such as actin-resistant DNase (alidornase-α) (288, 289) or DNase 1-like 3 (290) have been developed and some are currently being tested in phase I and II trials (288, 289). Macrophages from ARDS patients exhibit reduced capacity to clear NETs as well as apoptotic cells (263). Treatment of bronchoalveolar lavage fluid macrophages from ARDS patients *ex vivo* with the AMPK activator metformin (264) enhanced NET clearance and efferocytosis (263). In addition to blocking suicidal NET formation, T-series resolvins, and RvT2 in particular, were shown to facilitate NET uptake by monocyte-derived M0 macrophages *in vitro* as well as by peritoneal macrophages in mice (168), indicating the therapeutic potential of RvT2 in the *in vivo* setting.

## Concluding Remarks

Timely removal of neutrophils from the inflamed area is of outmost importance to efficient resolution of inflammation and return to homeostasis. Failure to clear neutrophils may lead to perpetuation of the inflammatory response and persisting tissue damage. Thus, exploring the fate of emigrated neutrophils and their contribution to mechanisms that distinguish self-limiting or protective inflammation from aggravated and chronic inflammation is critical to improve current therapies. The phenotypic heterogeneity and functional versatility of neutrophils and their diverse roles in innate and adaptive immune responses provide important cues for development of neutrophil-targeting therapies. However, whether the neutrophil`s actions are mediated by different polarization states of mature neutrophils or distinct neutrophil subsets remains unclear. A simple “one size fit all” anti-neutrophil approach is perhaps naïve and outdated (282). Indeed, over the past years numerous strategies have been developed, which show promising results in preclinical models to prevent the detrimental effects of neutrophils. These include molecules that can inhibit, restore or enhance specific neutrophil functions. Use of pro-resolving agonists, such as lipoxins, resolvins and annexin A1, which activate endogenous resolution programs and would serve as immunoresolvants rather than immunosuppressant (15), represent a conceptual change for the treatment of inflammatory pathologies as well as the emergence of “resolution pharmacology” (291). Although large-scale clinical studies with these compounds seem distant, some strategies, e.g. topical application of a LXA_4_ mimetic and degrading NETs within the lung with inhaled dornase-α, are currently being investigated in clinical trials. This ongoing research highlights the importance of targeting neutrophils, and distinct neutrophil subsets in particular, and will likely spur further advances in neutrophil-targeted therapies to dampen inflammation to favor reparative processes without comprising antimicrobial host defense.

## Author Contributions

The author confirms being the sole contributor of this work and has approved it for publication.

## Funding

This study was supported by grants from the Canadian Institutes of Health Research (MOP-97742 and MOP-102619).

## Conflict of Interest

The author declares that the research was conducted in the absence of any commercial or financial relationships that could be construed as a potential conflict of interest.

## Publisher’s Note

All claims expressed in this article are solely those of the authors and do not necessarily represent those of their affiliated organizations, or those of the publisher, the editors and the reviewers. Any product that may be evaluated in this article, or claim that may be made by its manufacturer, is not guaranteed or endorsed by the publisher.
